# Empirical Evaluation of Invariances in Deep Vision Models

**DOI:** 10.3390/jimaging11090322

**Published:** 2025-09-19

**Authors:** Konstantinos Keremis, Eleni Vrochidou, George A. Papakostas

**Affiliations:** MLV Research Group, Department of Informatics, Democritus University of Thrace, 65404 Kavala, Greece; kokerem@cs.duth.gr (K.K.); evrochid@cs.duth.gr (E.V.)

**Keywords:** invariances, vision transformers, convolutional neural networks, model robustness, image perturbations, deep learning, computer vision, artificial intelligence

## Abstract

The ability of deep learning models to maintain consistent performance under image transformations-termed invariances, is critical for reliable deployment across diverse computer vision applications. This study presents a comprehensive empirical evaluation of modern convolutional neural networks (CNNs) and vision transformers (ViTs) concerning four fundamental types of image invariances: blur, noise, rotation, and scale. We analyze a curated selection of thirty models across three common vision tasks, object localization, recognition, and semantic segmentation, using benchmark datasets including COCO, ImageNet, and a custom segmentation dataset. Our experimental protocol introduces controlled perturbations to test model robustness and employs task-specific metrics such as mean Intersection over Union (mIoU), and classification accuracy (Acc) to quantify models’ performance degradation. Results indicate that while ViTs generally outperform CNNs under blur and noise corruption in recognition tasks, both model families exhibit significant vulnerabilities to rotation and extreme scale transformations. Notably, segmentation models demonstrate higher resilience to geometric variations, with SegFormer and Mask2Former emerging as the most robust architectures. These findings challenge prevailing assumptions regarding model robustness and provide actionable insights for designing vision systems capable of withstanding real-world input variability.

## 1. Introduction

The ability of computational models to maintain consistent representations of visual data under geometric and photometric transformations—known as image invariances—has been a cornerstone of computer vision research. Effectively handling image invariances has profound implications across numerous practical and critical domains. Applications ranging from autonomous driving systems, robotic navigation, medical imaging diagnostics, surveillance and security systems to augmented reality heavily depend on robust invariant recognition mechanisms. Systems that inadequately manage invariances tend to exhibit significant performance deterioration when faced with minor deviations from idealized training conditions. Invariances ensure that a model recognizes an object regardless of its orientation (rotation invariance), size (scale invariance), or perturbations such as blur or noise [1,2]. For instance, a rotation-invariant system identifies a cat whether it is upright or upside-down, while scale invariance allows detection of the same object at varying distances. These properties are critical for real-world applications, where input data rarely conforms to idealized conditions.

Despite their importance, the mechanisms by which modern deep learning architectures such as convolutional neural networks (CNNs) and vision transformers (ViTs) achieve invariances remain poorly understood, particularly when compared to classical computer vision methods [3]. Moreover, systematic comparisons of invariances in CNNs and transformers are scarce. Prior work has focused on isolated transformations (e.g., rotation or scale) or specific architectures [3,4,5]. This work aims to empirically investigate how CNNs and transformers respond to systematic transformations, shedding light on their robustness and limitations. This paper bridges the identified gap in the literature by analyzing how both model families respond to injected invariances—controlled perturbations in rotation, scale, blur, and noise—against original images. By quantifying output deviations and latent space shifts, this work aims to provide a unified framework for evaluating invariances across model architectures. Our findings challenge assumptions about the inherent robustness of deep vision models and offer guidelines for designing more reliable vision systems. To this end, the main scope of this work is the design of a systematic benchmarking study providing the following contributions:

1. Unified evaluation across tasks—recognition, localization, and segmentation are rarely benchmarked together under identical perturbation protocols.
2. Controlled comparison—testing of 30 models under the same invariance transformations, ensuring a fair basis for comparison.
3. Empirical confirmation of assumptions—although prior works have suggested such robustness patterns, they were often task-specific or anecdotal. The presented results provide systematic evidence that these assumptions hold across different tasks and perturbations.

The rest of the paper is structured as follows: in Section 2, approaches to invariance are reviewed, from traditional to deep learning-based, as well as related works covering a wide range of invariances. Section 3 presents the proposed methodology, including datasets used, model selection and invariance applications. The experimental setup is described in Section 4, while the results are summarized in Section 5. Discussion and conclusions are included in Section 6 and Section 7, respectively.

## 2. Historical Review and Related Works

### 2.1. Historical Review

#### 2.1.1. Historical Approaches to Invariance

Before the advent of deep learning, achieving invariances required explicit algorithmic design. Early methods relied on handcrafted feature descriptors engineered to resist specific transformations. The Scale-Invariant Feature Transform (SIFT), introduced by Lowe (2004) [6], detected key points invariant to scale and rotation by analyzing gradient orientations in scale space [2]. These features were often paired with classifiers such as support vector machines (SVMs), which struggled with nonlinear decision boundaries in high-dimensional spaces [7]. For affine transformations, methods such as Affine-SIFT (ASIFT) extend SIFT by simulating multiple perspective deformations, despite their increased computational cost.

The popularity and effectiveness of SIFT spurred further refinements. One notable advancement in handcrafted invariant features was the Speeded-Up Robust Features (SURF), introduced by Herbert Bay et al. in 2006 [8]. SURF aimed to retain SIFT’s robustness while significantly improving computational speed. It utilized approximations of the Hessian matrix to quickly identify stable interest points and simplified box filters to approximate gradient orientations, thus substantially reducing computational overhead.

Additional methods such as ORB (Oriented FAST and Rotated BRIEF) [9] subsequently emerged, combining computational efficiency with robustness. ORB integrated the FAST (Features from Accelerated Segment Test) detector and BRIEF (Binary Robust Independent Elementary Features) descriptors, significantly improving feature detection speed. ORB became a valuable tool for applications requiring real-time processing on resource-limited devices, such as smartphones and embedded systems.

Alongside feature extraction techniques, preprocessing methods played a crucial role in achieving invariance. Prior to feature extraction, traditional systems applied various normalization procedures systematically to enhance robustness against specific image variations. Techniques such as histogram equalization and contrast adjustments were commonly employed to mitigate variations in illumination and enhance consistency in image appearance.

Another influential handcrafted method was the Histogram of Oriented Gradients (HOG) [10]. Unlike SIFT or SURF, HOG descriptors primarily focus on structured edge and gradient information throughout localized image blocks. While inherently less invariant, HOG demonstrated exceptional effectiveness for recognizing well-defined shapes, particularly for tasks such as pedestrian detection and surveillance monitoring.

Spatial Pyramid Matching (SPM) [11] provided yet another critical advancement. SPM extended the standard bag-of-features model by capturing spatial information through hierarchical spatial decomposition. Images were divided into grids at multiple scales, and local histograms of visual features were computed within each grid. This hierarchical structure offered partial invariance to translation and scale, significantly enhancing the discriminative power of classification systems by preserving essential spatial layout information

*Scale invariance* represents one of the fundamental challenges in computer vision, requiring feature descriptors to remain consistent across different image scales. The mathematical foundation for scale invariance was primarily addressed through two complementary approaches: transform-domain methods and multi-scale representations. The Mellin transform [12] provided a theoretical foundation for scale invariance through its mathematical properties. The transform is defined as:

(1) $$M[f](s)=\int_{0}^{\infty }\;x^{s-1}f(x)dx$$

where f(x) is a function defined on (0, ∞), *s* is a complex value and the kerel $x^{s-1}$ denotes the transformation of its multiplicative convolution nature. For a scaled function g(x)=f(kx), the relationship between the original and scaled transforms demonstrates a predictable scaling behavior:

(2) $$\left|M[g](s)|=|k^{-s}||M[f](s)\right|$$

where *k* is a positive constant. While this relationship shows that the magnitude is not perfectly invariant, it establishes a mathematically tractable connection that enables scale normalization through logarithmic operations. The scaling factor k can be extracted using $log\frac{g(s)}{f(s)}$ for each integral coefficient, providing a mechanism for scale calibration in feature matching applications.

The theoretical treatment of *blur invariance* assumed circularly symmetric point spread functions (PSFs) represented as *h*(*r*) in polar coordinates. For a blurred image *g*(*r,θ*) = *f*(*r*,*θ*)∗*h*(*r*), the Zernike moments exhibit a complex linear relationship established by Chen et al. [13]:

(3) $$Z_{n,m}^{\left(g\right)}=\sum_{i=0}^{l}\;Z_{n-2i,m}^{\left(f\right)}\sum_{j=0}^{l-i}\;Z_{2j,0}^{\left(h\right)}A(m,l,i,j)$$

where $Z_{2j,0}^{\left(h\right)}$ represents the Zernike moments of the circularly symmetric PSF, and A(m,l,i,j) are coefficient matrices that depend on the mathematical properties of the radial polynomials. This fundamental relationship enabled the derivation of combined blur-geometric invariants through moment ratio formulations and recursive construction of blur-invariant descriptors.

*Rotation invariance* was primarily achieved through mathematical invariants derived from image moments, with Hu moments [14] representing the most widely adopted approach for shape-based feature extraction. Hu moment invariants provide a set of seven mathematical descriptors that remain constant under rotation transformations. These invariants are derived from geometric moments, which are calculated as:

(4) $$m_{pq}=\sum \sum x^{p}y^{q}f(x,y)$$

where p and q are non-negative integers defining the moment order, and f(x,y) is the image intensity function. The central moments, which provide translation invariance, are computed relative to the centroid:

(5) $$\mu_{pq}=\sum \sum (x-\bar{x}{)}^{p}(y-\bar{y}{)}^{q}f(x,y)$$

The seven Hu moment invariants are constructed through specific combinations of normalized central moments, providing mathematical guarantees of rotation invariance while maintaining discriminative power for shape analysis. These invariants have been empirically validated for shape matching applications and demonstrate robust performance across rotational transformations.

Zernike moments offer an alternative mathematical framework for rotation invariance through orthogonal polynomial representations. The complex Zernike moments [15] are defined using polynomials that form a complete orthogonal basis set on the unit disc:

(6) $$Z_{nm}=\frac{n+1}{\pi }\int \int_{x^{2}+y^{2}\leq 1}\;f(x,y)[V_{nm}(x,y){]}^{*}dxdy$$

where,  f(x,y) is the image intensity function, and $V_{nm}(x,y)$ is the Zernike polynomial of order *n* and repetition *m*. The Zernike formulation demonstrates superior performance compared to alternative moment-based approaches in terms of noise resilience, information redundancy, and reconstruction capability. The orthogonal nature of the basis functions ensures minimal information overlap between different moment orders, providing efficient and robust feature representations for rotation-invariant applications. The translation-invariant central moments are computed using the centroid coordinates $\left(\bar{x},\bar{y}\right)$, $\bar{x}=\frac{m_{10}}{m_{00}},\bar{y}=\frac{m_{01}}{m_{00}}$. This mathematical framework ensures that shape descriptors remain consistent regardless of the object’s position within the image frame. The zeroth-order moment $m_{00}$ represents the total intensity or area of the region, providing a normalization factor for higher-order moment calculations.

**Figure 1:**
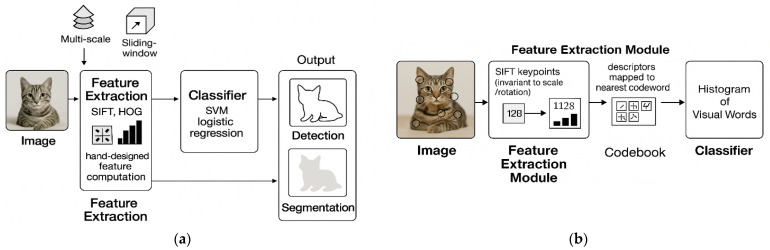
Handling invariances with traditional feature extraction using: (**a**) handcrafted features; (**b**) Bag of Words (BoW).

A significant limitation of these approaches was their reliance on domain expertise to predefine invariants. For example, Fourier-Mellin moments [16] provided rotational invariance by projecting images into a polar coordinate system, but their efficacy depended on careful parameter tuning [17]. Additionally, traditional methods faced challenges in generalizing across diverse datasets, as manually engineered features often failed to capture the full complexity of natural image variations [7]. The introduction of data augmentation—applying random transformations to training data—partially addressed these issues by exposing models to broader input distributions [18]. However, augmentation strategies were heuristic and did not guarantee invariance at test time [18]. Figure 1 portray traditional ways of handling image invariances before the advent of deep learning.

#### 2.1.2. The Deep Learning Shift

The rise of CNNs in the 2010s marked a paradigm shift, as hierarchical architectures automatically learned invariant features through stacked convolutional and pooling layers [5]. Krizhevsky et al. [19] demonstrated that max-pooling in CNNs could induce translational invariance by discarding spatial information, while deep layers captured increasingly abstract patterns. Subsequent research works explored equivariant networks, which preserved transformation relationships across layers through structured weight-sharing [3,4]. For instance, steerable CNNs enforced rotational equivariance by constraining filters to harmonic basis functions, enabling precise control over invariance properties [4].

**Figure 2:**
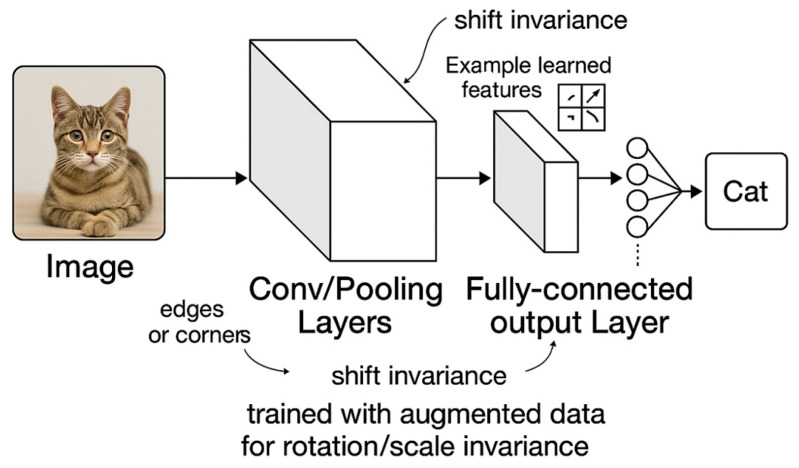
Pipeline for handling invariances through deep learning.

Transformers, initially developed for natural language processing, introduced self-attention mechanisms that dynamically weighted input patches based on global context [20]. Vision transformers (ViTs) achieved state-of-the-art performance by treating images as sequences of patches, but their invariance mechanisms differed fundamentally from CNNs [5,20]. Unlike convolutional inductive biases, ViTs relied on learned positional embeddings, raising questions about their robustness to geometric distortions. Recent studies highlight that while CNNs exhibit local translational invariance, their global invariance remains imperfect, with small pixel shifts sometimes drastically altering predictions [5,21]. In Figure 2, a simplified portrayal of how image invariances are handled in deep learning is shown.

#### 2.1.3. Emerging Trends in Self-Supervised Learning for Invariance

Recent advances in self-supervised learning have revolutionized how invariances are learned in deep models. Contrastive learning approaches like SimCLR and MoCo-v2 implicitly encode invariances through data augmentation strategies, creating positive pairs that force the model to develop robust representations [7]. These methods demonstrate that the choice of augmentations directly determines which invariances the model acquires, with implications for downstream task performance. Ruchika et al. [22] introduced the concept of amortized invariance learning, where the feature extractor was parameterized by differentiable invariance hyper-parameters that could be efficiently adapted to match task-specific invariance requirements. This approach provides flexibility without requiring expensive re-training for each downstream application. However, such methods must carefully balance invariance learning against the risk of discarding semantically meaningful features, especially when aggressive augmentation strategies are employed [2,17]. The integration of null space-based noise augmentation during fine-tuning has shown promise in improving resilience to distribution shifts while preserving key visual attributes [2].

#### 2.1.4. Domain Invariance as a Path to Robustness

The connection between invariance learning and adversarial robustness has emerged as a critical research direction. Traditional adversarial training methods have been extended to incorporate domain invariance objectives, as demonstrated by DIAL (Domain Invariant Adversarial Learning), which constrains feature representations to be indistinguishable between natural and adversarial perturbed examples [23]. This approach not only enhances robustness against adversarial attacks but also improves performance on natural distribution shifts. Similarly, DAT (Domain-wise Adversarial Training) leverages domain-specific perturbations to eliminate spurious correlations that hinder out-of-distribution generalization [24]. For vision transformers, which have shown superior robustness compared to CNNs, recent work has identified that they still rely heavily on non-robust features surviving patch-based transformations [25]. Regularization techniques using patch-based negative augmentations have effectively mitigated this dependency, consistently improving performance on robustness benchmarks such as ImageNet-C and ImageNet-R without sacrificing in-distribution accuracy [26]. These findings highlight the importance of considering both positive augmentations (teaching models to be insensitive to spurious changes) and negative augmentations (preventing reliance on non-robust features) to achieve optimal robustness in deep vision models.

### 2.2. Related Works

#### 2.2.1. Blur Invariances

Blur invariance represents a model’s ability to maintain consistent performance when processing images affected by various types of blurring distortions. Blur corruptions significantly degrade the performance of image recognition systems by removing high-frequency details and edge information crucial for accurate classification. Recent studies have demonstrated that blurring effects, alongside other common image distortions such as noise and compression artifacts, substantially elevate error rates in neural networks [27]. This vulnerability stems from the models’ inherent architecture designs, which typically lack explicit mechanisms to handle frequency domain perturbations, making them particularly susceptible to low-frequency corruptions (LFc), like blur, that distort critical spatial information [28].

Several approaches have been proposed to address blur invariance in CNNs in the past decade. Anti-aliasing techniques have been proven effective, using low-pass filtering before down-sampling operations to make convolutional networks more shift-invariant and subsequently more robust to distortions [29]. AugMix, proposed by Hendrycks et al. [30], demonstrated significant improvements in robustness through a data processing technique that diversifies the training distribution with various corruptions, including blur. Similarly, Adversarial Augment optimizes image-to-image transformations to generate adversarial corrupted training examples, strengthening model resilience to common corruptions [31]. More frequency-aware approaches include LP-ReLU, which instills low-pass filtering directly into activation functions, complemented by Discrete Cosine Transform (DCT) augmentation to specifically target both high and low-frequency corruptions [28]. The self-training method with Noisy Student [32] has also shown remarkable improvements on robustness benchmarks such as ImageNet-C, reducing mean corruption error from 45.7 to 28.3.

ViTs have demonstrated different vulnerability patterns compared to CNNs when facing blur corruption. Recent analysis revealed that transformer-based vision models show particular sensitivity to blur and snow corruptions, which the authors connect to transformers’ inherent bias toward low-frequency processing [33]. To enhance ViTs’ robustness, Herrmann et al. [34] proposed Pyramid Adversarial Training (PyramidAT), which achieves state-of-the-art performance on robustness benchmarks including ImageNet-C, ImageNet-R, and ImageNet-Sketch without requiring additional training data. Spatial Prior-enhanced Vision Transformers (SP-ViT) introduce learned spatial inductive biases that consider various spatial relations, potentially offering improved resilience to spatial distortions [35]. Comparative studies across architectures indicate that ViTs trained on extensive datasets like JFT-300M exhibit notable resilience to various distortions, including blur [27]. For evaluation, researchers have developed specialized benchmarks including ImageNet-C for common corruptions, TextVQA-C and GQA-C for assessing how corruptions affect visual reasoning tasks, and most recently, ImageNet-D, which leverages diffusion models to generate challenging test images with diverse backgrounds, textures, and materials [33,36].

#### 2.2.2. Noise Invariances

Noise invariance refers to a neural network’s ability to maintain performance when inputs are corrupted with noise. Additive noise, particularly Gaussian noise, disrupts the feature extraction process in CNNs by altering pixel values, leading to misclassifications in real-world scenarios. Research addressing this challenge has explored multiple strategies: data augmentation techniques like AugMix [30] improve robustness with minimal computational overhead; stability training [37] encourages consistent outputs despite input perturbations; feature denoising [38] integrates filtering operations directly into network architectures to clean feature maps. Cohen et al. [39] advanced randomized smoothing by adding Gaussian noise during both training and inference, achieving 49% certified accuracy on ImageNet under adversarial perturbations. These approaches demonstrate that dedicated noise-handling mechanisms can significantly improve CNN robustness.

ViTs have recently demonstrated superior robustness to noise compared to CNNs. Paul et al. [40] found that ViTs exhibit significantly better robustness against distribution shifts and common corruptions, attributing this to reduced sensitivity to high-frequency perturbations. To standardize evaluation, Hendrycks and Dietterich [41] introduced ImageNet-C and ImageNet-P benchmarks for testing performance against corruptions and perturbations. These benchmarks revealed that while classification accuracy has improved from AlexNet to modern architectures, relative robustness to corruptions has seen negligible improvements. This suggests that noise invariance requires dedicated strategies beyond architectural innovations, an insight applicable to both CNNs and ViTs in ensuring reliable performance in noisy real-world environments.

#### 2.2.3. Rotation Invariances

Rotation invariance refers to a model’s ability to recognize objects regardless of their orientation in an image. While CNNs inherently possess translational invariance through weight sharing across spatial locations, they lack intrinsic rotational invariance. This limitation means standard CNNs often perform poorly when encountering objects at orientations different from those seen during training. As demonstrated in multiple studies, classification accuracy can drop dramatically when images are rotated, requiring models to either learn all possible rotations through extensive data augmentation or employ specialized architectures to address this fundamental challenge [42].

Several approaches have emerged to address rotation sensitivity in CNNs. Spatial Transformer Networks (STNs) [43] pioneered the concept of learned spatial manipulations within the network, enabling models to actively transform feature maps based on input content. Group Equivariant CNNs (G-CNNs) [44] extended conventional convolutions to be equivariant to discrete rotations and reflections, achieving state-of-the-art results on rotated MNIST with substantially increased weight sharing. Harmonic Networks [45] implemented rotation equivariance by replacing standard filters with circular harmonics, producing orientation-aware feature maps without increasing parameter count. Alternative approaches include PROAI (Pre-Rotation Only At Inference time) [46], which trains on samples at a single orientation but performs inference across multiple rotated versions of test samples, significantly reducing training complexity while maintaining competitive performance on rotated MNIST benchmarks.

More recently, ViTs have demonstrated potential for improved rotation robustness. The Rotation Invariant Vision Transformer (RViT) [47] incorporates rotated patch embeddings to enhance performance in medical imaging tasks, achieving superior metrics (sensitivity 1.0, specificity 0.975) compared to standard ViTs. IrisFormer [48] employs relative positional encoding rather than absolute positional encoding to achieve rotation invariance, complemented by horizontal pixel-shifting during training. The Artificial Mental Rotation (AMR) [49] approach, applicable to both CNNs and ViTs, outperforms rotational data augmentation by 19% when evaluated across ImageNet, Stanford Cars, and Oxford Pet datasets. Rotation-Invariant Transformer for UAV object recognition [50] simulates rotation at the patch feature level rather than through image-level augmentation, achieving 5.9% and 4.8% improvements in mAP and Rank1 metrics, respectively. These advances demonstrate the evolving landscape of rotation-invariant deep learning approaches, with each architecture offering distinct trade-offs between computational efficiency, parameter count, and rotation robustness.

#### 2.2.4. Scale Invariances

Scale invariance in image recognition refers to a model’s ability to correctly identify objects regardless of their size or the image’s resolution. Standard CNNs often struggle with this property due to their fixed receptive field sizes and architectural constraints that limit their ability to handle objects appearing at different scales [51,52]. This limitation is particularly evident when CNNs trained on objects of specific sizes encounter significantly larger or smaller instances during inference. While CNNs can implicitly learn some degree of scale invariance through data augmentation, they lack the intrinsic mechanisms to effectively model scale variations across wide ranges without explicit architectural considerations [51]. The absence of robust scale invariance can be particularly problematic in domains such as medical imaging, where the physical size of objects (e.g., tumors, lesions) carries crucial diagnostic information.

Recent research in the last decade has proposed various architectural modifications to enhance scale invariance in CNNs. Spatial Pyramid Pooling (SPP) has emerged as a prominent approach, where features are extracted at multiple scales and then aggregated to improve robustness to scale variations [53,54]. Multi-scale feature fusion techniques have been implemented in models like MFP-CNN, which effectively capture information at different scales to mitigate intra-class diversity challenges [53]. The Feature Map Augmentation approach introduces variations in features rather than input data, with methods like the Rotation Invariance Transformer (RiT) applying transformations to augment CNN features for improved scale invariance with minimal computational overhead [55]. Deep Spatial Pyramid Ensemble frameworks have demonstrated effectiveness in event recognition tasks by building universal representations from multiple scales [56]. Additionally, the SFCNN architecture implements a pyramid of stacked filters of different sizes to extract features from larger spatial areas, drawing inspiration from the human visual system’s global-first response strategy [55,57].

Vision Transformers have introduced new paradigms for handling scale invariance through their attention mechanisms and hierarchical designs. However, traditional Transformers lack inherent inductive biases for modeling local visual structures and scale variance, requiring specific adaptations [58,59]. ECViT addresses this limitation by combining CNN strengths with Transformer capabilities, incorporating a pyramid structure for efficient multi-scale feature extraction and representation [60]. The ViTAE architecture employs spatial pyramid reduction modules to embed input images into tokens with rich multi-scale context, thereby acquiring intrinsic scale invariance properties [58]. EViTIB integrates CNN inductive biases into Transformers by incorporating a parallel convolution branch alongside the self-attention mechanism to better model local patterns across scales [61]. RPViT takes a different approach by extracting regions with locality using traditional region proposal algorithms and normalizing objects of different scales to the same size through bilinear interpolation [59].

## 3. Materials and Methods

**Figure 3:**
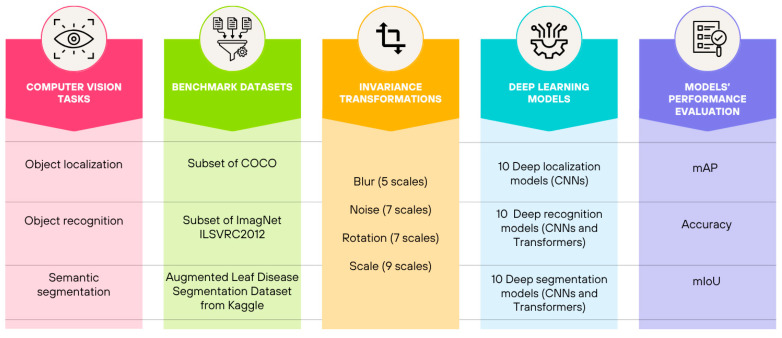
Pipeline of the proposed methodology towards studying deep learning model invariances.

The proposed approach is illustrated in Figure 3. Three key computer vision tasks are examined: object localization, object recognition, and semantic segmentation. For each task, a benchmark dataset is selected, and four invariance transformations are applied to images, towards evaluating, through targeted performance metrics, the robustness of 10 different deep learning models for each task, to applied invariances.

The rest of the section presents a detailed overview of the datasets utilized for evaluating models’ performance (ten models for each task) across the three key computer vision tasks. To ensure robust comparative analysis, we carefully selected established benchmark datasets while addressing the need for consistent evaluation metrics through strategic dataset preparation and fine-tuning approaches.

### 3.1. Benchmark Datasets

The empirical evaluation required datasets that could effectively assess model invariances across different vision tasks. The datasets were selected based on their prevalence in the literature, quality of annotations, and suitability for our specific evaluation needs.

#### 3.1.1. Object Localization Dataset

For evaluating localization models, we utilized a subset of the COCO validation dataset. The COCO (Common Objects in Context) dataset is widely recognized for its complex scenes containing multiple objects at various scales with detailed instance-level annotations [62]. All localization models in our study were originally trained on the COCO dataset, making its validation set an appropriate choice for evaluation.

This selection was necessitated by the lack of available test data with corresponding ground truth labels. The COCO validation set contains extensive instance annotations, including bounding boxes and segmentation masks across 80 common object categories [62,63]. This rich annotation scheme enabled us to evaluate fine-grained localization performance across different scales and object configurations. The original size of the dataset consisted of 5000 different images, yet a smaller random selection of five images for each class was made, 400 images in total. The COCO validation subset provided sufficient diversity to assess model invariance to object scale, perspective, occlusion, and background complexity, which are considered key aspects in our investigation of CNN invariances.

#### 3.1.2. Object Recognition Dataset

For recognition models, we employed a carefully selected subset of the ImageNet ILSVRC2012 validation dataset. The ImageNet Large Scale Visual Recognition Challenge 2012 (ILSVRC2012) provides a robust benchmark for object recognition with 1000 object categories [64,65]. The dataset consists of thousands of images, but our subset was randomly selected to provide a sample of five images per class with a total of 5000 images.

This subset was chosen primarily because it represents the de facto standard for training and evaluating recognition models. The diverse nature of ImageNet categories allowed us to assess recognition performance across a wide spectrum of object types, illumination conditions, and viewpoints. This diversity was crucial for our study of CNNs’ invariances in the recognition domain. The validation subset contains high-quality annotations and presents challenging recognition scenarios that test the limits of model invariance to various transformations and real-world conditions.

#### 3.1.3. Semantic Segmentation Dataset

To address the challenge of comparing models trained on different datasets, we incorporated a custom dataset retrieved from Kaggle [66]. This approach was necessary because the segmentation models in our study were originally trained on different datasets with varying annotation schemes and object categories. This dataset was used for fine-tuning the already trained models. Fine-tuning generally requires much less data, since the model already has rich learned representations. Even a few thousand samples can be enough to adapt a pre-trained model to a new domain. An important reason for its selection was that its data is always centered. The original dataset contained 588 pictures of leaves with a type of disease on them, together with their annotation. After reviewing the dataset and removing unsuitable photos, an augmentation was made to reach a more appropriate number of 3138 images that were used for the fine-tuning of each model, while 62 images from the original dataset were kept intact for the inference and validation of the models. Augmentation included the rotation of images by 60 degrees in order to create a more diverse dataset. Finally, the decision to use a small subsample of data was made due to our limited computational resources.

The custom dataset served as a crucial fine-tuning benchmark, particularly for transformer-based segmentation models. By fine-tuning all models on this common dataset, we established a more equitable baseline for performance comparison, mitigating the bias introduced by differences in pre-training data [64,67].

### 3.2. Models’ Selection Criteria

The selection criteria prioritized models that are openly accessible, widely adopted within the research community, and extensively documented online. More specifically, selection criteria included the following aspects:

- *Documentation and Resource Availability*. The most important factor for model selection was firstly models that were trained on open source and well-constructed datasets, and secondly models with comprehensive documentation, tutorials, and implementation examples were favored to facilitate integration into our research pipeline. The availability of educational resources surrounding these models ensures efficient troubleshooting and optimization.
- *Open-Source Availability*. All selected models need to be available through open-source licenses, allowing for unrestricted academic use and modification. This accessibility is crucial for reproducibility and extension of our research findings. Open-source models also typically provide pre-trained weights on standard datasets, reducing the computational resources required for implementation. Models with significant adoption within the computer vision community were prioritized. All models were imported from well-known libraries such as PyTorch or Hugging Face.
- *Performance-Efficiency Balance*. This criterion spans a range of architectures that offer different trade-offs between accuracy and computational efficiency. This variety allows us to evaluate which models best suit specific hardware constraints and performance requirements. Many models had the ability to select multiple versions of each other with different sizes of parameters, which made them much easier or much harder to run. For this research, each model was selected based on the ability of the testing hardware to infer it in the shortest amount of time without reducing the performance due to its small number of parameters.

Table 1, Table 2 and Table 3 include the selected models, ten for each one of the three key computer vision tasks: object localization, object recognition, and semantic segmentation.

At this point, it should be noted that all models evaluated for localization and recognition were pretrained on their respective standard datasets—localization models on COCO and recognition models on ImageNet—consistent with common practice and the model authors’ original training regimes. However, for the semantic segmentation task, the selected models originated from diverse training backgrounds, having been trained on different datasets with varying annotation schemas. In the latter case, to ensure a fair and consistent comparison across segmentation architectures, we standardized their evaluation by fine-tuning all segmentation models on the same custom dataset

### 3.3. Generating Degraded Images

#### 3.3.1. Blurred Images

To assess the robustness of the models under image blur distortions, we applied Gaussian blurring at multiple levels to a fixed subset of images from the dataset. The blurring was implemented using a Gaussian kernel of fixed size (5 × 5), with the degree of blur controlled by varying the standard deviation (σ, or sigma) parameter. Specifically, we evaluated blur invariance across five sigma values: σ = 0 (no blur), 1, 2, 3, and 4. The application of Gaussian blur was performed using OpenCV’s GaussianBlur function, with identical values for both sigmaX and sigmaY to ensure isotropic smoothing.

Unlike geometric transformations, the blur operation does not require adjustment of bounding box coordinates, as it preserves the spatial layout of the image. For each level of blur, the blurred images were passed directly to a pre-trained model for object detection. Model predictions were then evaluated against the unaltered ground truth annotations.

#### 3.3.2. Noised Images

To investigate the robustness of the models to additive Gaussian noise, we applied varying levels of pixel-wise noise to images of the dataset. Gaussian noise was synthetically added to each image in the normalized pixel intensity range [0, 1]. Specifically, noise was sampled from a zero-mean normal distribution with standard deviations (std) of 0.0, 0.05, 0.1, 0.15, 0.2, 0.25, and 0.3. The noise was added to the RGB image values, and the results were clipped to maintain valid pixel intensity bounds. Afterwards, the images were scaled back to the standard matching range of the input for the model.

This transformation preserved the original spatial and structural layout of the images and did not require any modification to ground truth bounding boxes. For example, the same annotations provided by the COCO dataset were used for performance evaluation across all noise levels. Inference was performed on each noise-augmented image using a pre-trained model. The model outputs were compared against the ground truth annotations.

#### 3.3.3. Rotated Images

To evaluate the robustness of the models under rotation transformations, we systematically applied controlled image rotations to the datasets. For each selected image, a series of rotations was performed at fixed angular intervals, specifically from 0 to 330 degrees. Yet, it should be noted that for the experiments, only up to 180 degrees were used, since beyond this range, the same results are mirrored, offering no additional insights. Moreover, inclusion of redundant degree points would significantly increase the models’ runtime without further contributing meaningful results. The rotation was applied using a standard 2D affine transformation centered at the image midpoint. To ensure that the entire rotated image content was preserved within the frame, the dimensions of the output image were adjusted based on the rotation matrix to accommodate any spatial expansion resulting from the transformation.

Crucially, for the case of the localization task, ground truth bounding boxes associated with each image were also transformed to align with the rotated image coordinates. This was achieved by computing the rotated positions of the four corners of each bounding box and then recomputing the axis-aligned bounding box that minimally enclosed these rotated points. This process ensured that object annotations remained consistent and accurate under all transformation conditions. Following the rotation, the transformed images and their adjusted annotations were input into the appropriate model.

#### 3.3.4. Scaled Images

To evaluate the scale invariance of the models, we conducted a series of controlled image resizing operations on the datasets. Each image was rescaled using a range of predefined scale factors: 0.1, 0.25, 0.5, 0.75, 1.0 (original resolution), 1.25, 1.5, 2.0, and 3.0. The scaling was performed using bilinear interpolation via OpenCV’s resize function, ensuring consistent image quality across resolutions.

After rescaling, each image was passed through each model. Since the resized images modified the spatial scale of objects, all predicted bounding boxes were rescaled back to the original coordinate frame before evaluation. This normalization step ensured that detection results could be directly compared against the original ground truth annotations provided by the dataset.

**Figure 4:**
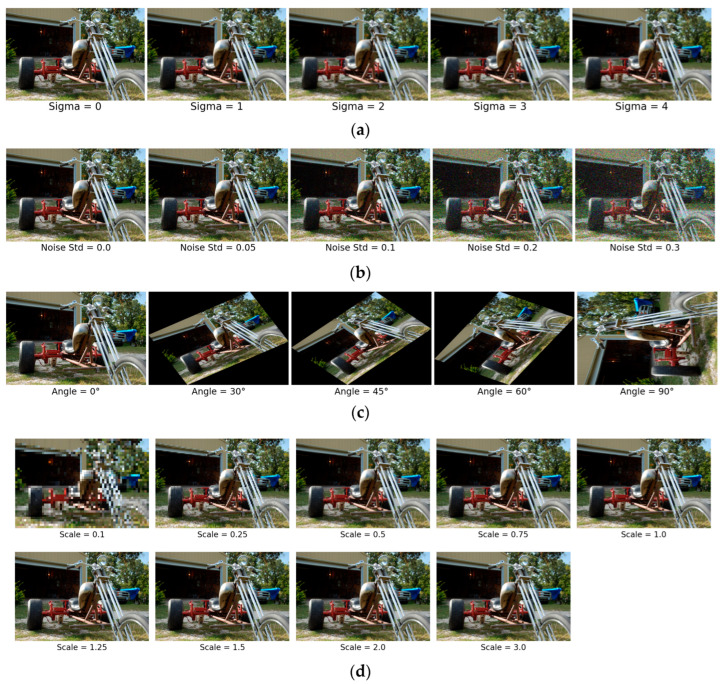
Indicative image transformations: (**a**) Gaussian blur of different strengths; (**b**) Gaussian noise of different strengths; (**c**) Rotation of different angles; (**d**) Scaling.

Figure 4 includes indicative visualizations of an image passing through different levels of blur (Figure 4a), Gaussian Noise (Figure 4b), Rotation (Figure 4c), and Scaling (Figure 4d). Note that the figures are provided for reference and do not depict all possible scales of invariances.

### 3.4. Models’ Performance Evaluation

A comprehensive evaluation framework was employed to assess model performance across localization, recognition, and segmentation tasks, followed by a systematic robustness analysis against image perturbations. The methodology aligns with recent advances in vision model evaluation while addressing domain-specific requirements through tailored metric selection and transformation protocols.

Localization performance was quantified using the Mean Average Precision (mAP). mAP is calculated across Intersection-over-Union (IoU) thresholds ranging from 0.50 to 0.95 in 0.05 increments, following the COCO evaluation protocol, and provides a robust measure of detection accuracy across various overlap criteria [68].

Recognition performance was assessed by using Accuracy, calculated as: Accuracy = (*TP + TN*)/(*TP + TN + FP + FN*), where *TP*, *TN*, *FP*, and *FN* refer to true positive, true negative, false positive and false negative, respectively. Accuracy measures the overall correctness of classification across all classes [69].

Finally, we evaluated segmentation performance by using the Mean Intersection over Union (mIoU). Calculated as: mIoU = (1/*C*) *× Σ* [*TP_c*/(*TP_c + FP_c + FN_c*)] for *c* = 1 to *C*, where *C* is the number of classes, *TP_c* is the number of true positives for class *c*, *FP_c* is the number of false positives, and *FN_c* is the number of false negatives. This metric penalizes both over- and under-segmentation errors while being class-agnostic.

This multi-metric approach ensured comprehensive characterization of model capabilities, with mIoU emphasizing structural segmentation fidelity, mAP quantifying localization reliability, and Accuracy reflecting diagnostic recognition accuracy under real-world conditions [70,71]. The unified evaluation framework enabled direct cross-model comparisons while maintaining alignment with domain-specific performance requirements for plant disease analysis.

## 4. Experimental Setup

To ensure a fair and statistically meaningful assessment of model performance under variant transformations, we selected five random images per class for both localization and recognition models. This sampling strategy strikes a balance between computational efficiency and representational diversity, enabling a robust estimation of performance without overwhelming the evaluation pipeline. By drawing multiple images per class, we mitigate the risk of bias that might arise from atypical examples or outliers, thereby improving the generalizability of the reported metrics. This approach also facilitates class-wise performance comparisons and supports a more granular analysis of invariance effects across different object categories.

For the inference, both the localization and recognition models were evaluated under controlled variations. Although a larger sample size could further stabilize the statistical estimates, the chosen five-image-per-class configuration provides sufficient coverage for detecting trends and patterns in model robustness, especially when compounded over many classes. This design also allows us to compute average metrics and confidence intervals, which are essential for drawing reliable conclusions about model sensitivity to transformations.

The experimental evaluation of the segmentation models was conducted using a custom dataset of diseased leaf images obtained from Kaggle. The initial dataset was relatively small, comprising paired images of diseased leaves along with their corresponding segmentation masks that precisely delineate the affected regions. To address the limited size of the original dataset and ensure sufficient training data for effective model fine-tuning, a systematic data augmentation strategy was implemented. Each original image was subjected to three successive 90-degree rotations, effectively quadrupling the dataset size by creating four distinct variations of each source image (the original plus three rotated versions). This geometric transformation-based augmentation approach was selected to preserve the essential structural characteristics of leaf diseases while introducing sufficient variability to enhance model generalization capabilities [72,73]. The augmentation protocol maintained the paired relationship between input images and their corresponding segmentation masks, ensuring consistency in the training data.

For all ten architectures—whether convolutional or transformer-based—we followed a uniform fine-tuning pipeline on our custom leaf dataset: we first wrapped the raw RGB images and their binary masks into a PyTorch Dataset, applying identical spatial preprocessing (resizing to the input of the model, normalizing inputs, thresholding masks to {0,1}) and retaining originals for visualization; next, we fed each sample through the respective model’s built-in preprocessing or a shared image processor to produce tensor inputs, then reconfigured the model’s classification head to output two classes (background vs. leaf), allowing weight adaptation via an “ignore mismatched sizes” flag when remapping final layers. During training, we batched data with a custom collate function, back-propagated a pixel-wise segmentation loss across the entire network and monitored validation IoU for early stopping.

## 5. Results

This comprehensive empirical study presents systematic evaluation results of deep learning models across three computer vision tasks—object localization, recognition, and semantic segmentation—under four types of invariance transformations. The evaluation encompasses 30 distinct models tested against blur, noise, rotation, and scale invariances using established benchmark datasets, including COCO validation subset, ImageNet ILSVRC2012 validation subset and the augmented custom Kaggle leaf disease segmentation dataset. Our findings reveal significant variation in patterns of robustness across model architectures and transformation types, providing empirical evidence for the current limitations of invariance handling in modern deep learning systems.

### 5.1. Object Localization

**Figure 5:**
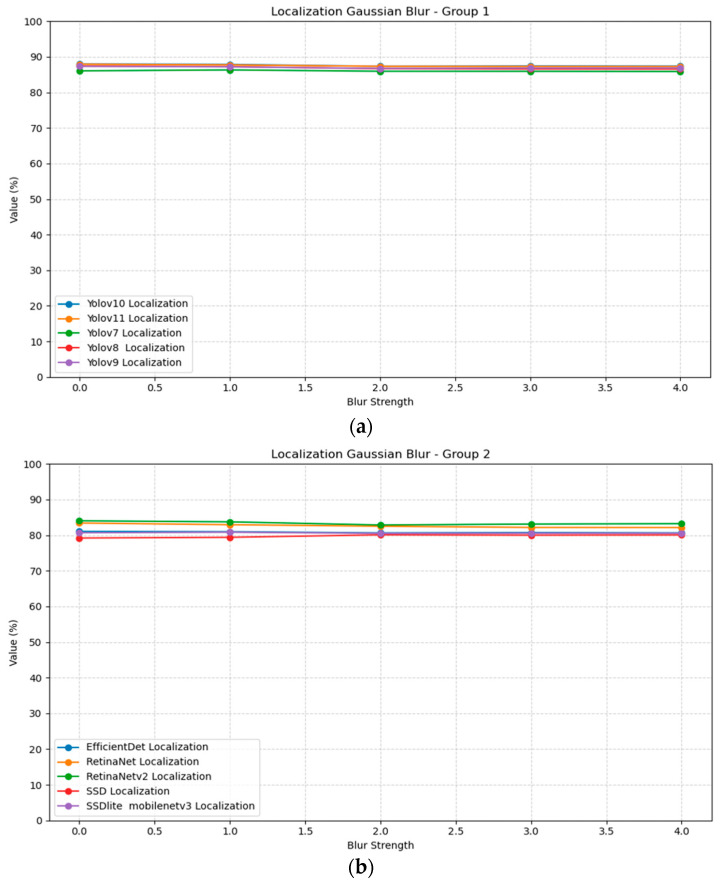
Localization Gaussian blur plots for all models and strengths: (**a**) Group 1; (**b**) Group 2.

The evaluation of object localization models under Gaussian blur transformations reveals varying degrees of robustness across different architectures. Table 4 presents the mean Average Precision (scores for ten localization models tested with Gaussian blur kernels ranging from 0 (no blur) to 4. YOLOv10 and v11 demonstrate the highest baseline performance at 0.88 mAP with sigma = 0, followed closely by YOLOv8 and v9 at 0.87. Notably, we found that most modern localization models exhibit strong robustness to Gaussian blur, maintaining mAP within ±0.01 across blur strengths up to sigma = 4. This suggests that coarse object structure is sufficient for accurate localization in these cases. Figure 5 illustrates the localization blur plots for all models, divided into two groups for better visualization; Group 1 includes Yolov10, Yolov11, Yolov7, Yolov8, and Yolov9, while Group 2 includes EfficientDet, RetinaNet, RetinaNetv2, SSD, and SSDlite.

**Figure 6:**
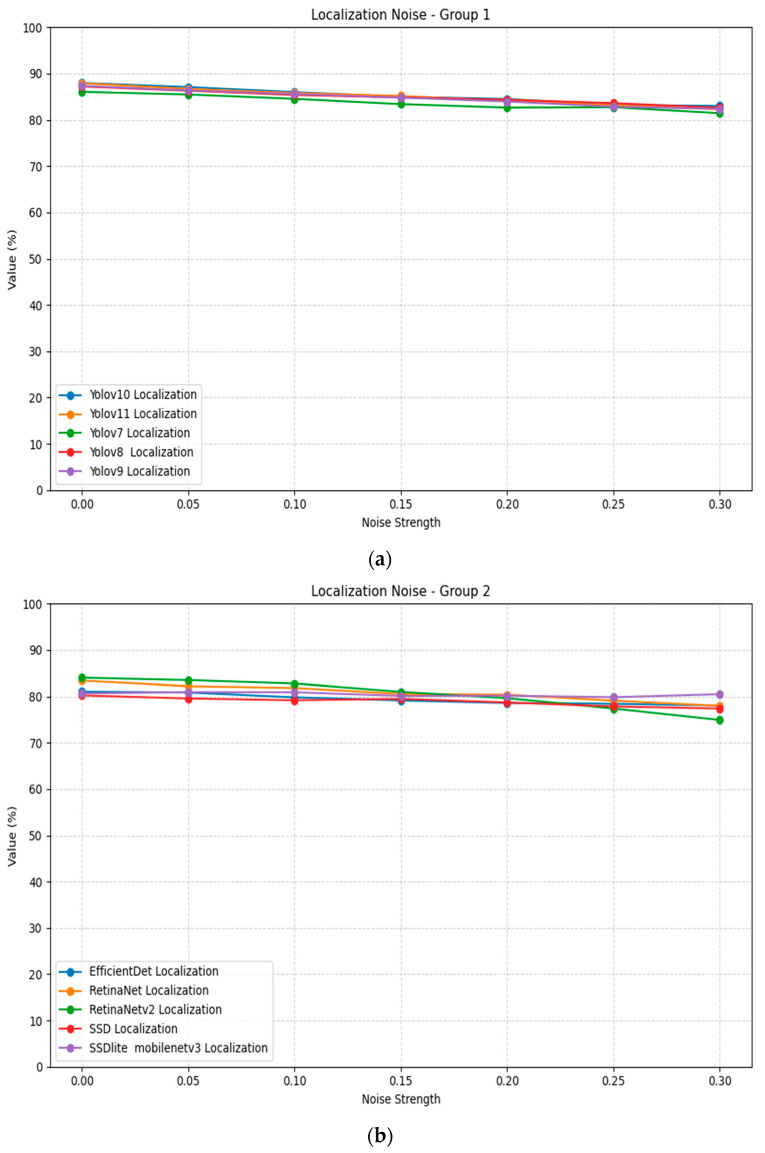
Localization Gaussian noise plots for all models and strengths: (**a**) Group 1; (**b**) Group 2.

Gaussian noise impacts localization performance across all tested models to a higher percentage, as evidenced in Table 5. The results demonstrate a consistent pattern of performance degradation as noise intensity increases from 0 to 0.3. RetinaNetv2 shows the most severe degradation, dropping from 0.84 at std = 0 to 0.75 mAP at std = 0.3, representing a sizable performance loss. Most models manage to keep an acceptable performance even in higher amounts of noise. Figure 6 illustrates the localization noise plots for all models, divided into two groups for better visualization.

**Figure 7:**
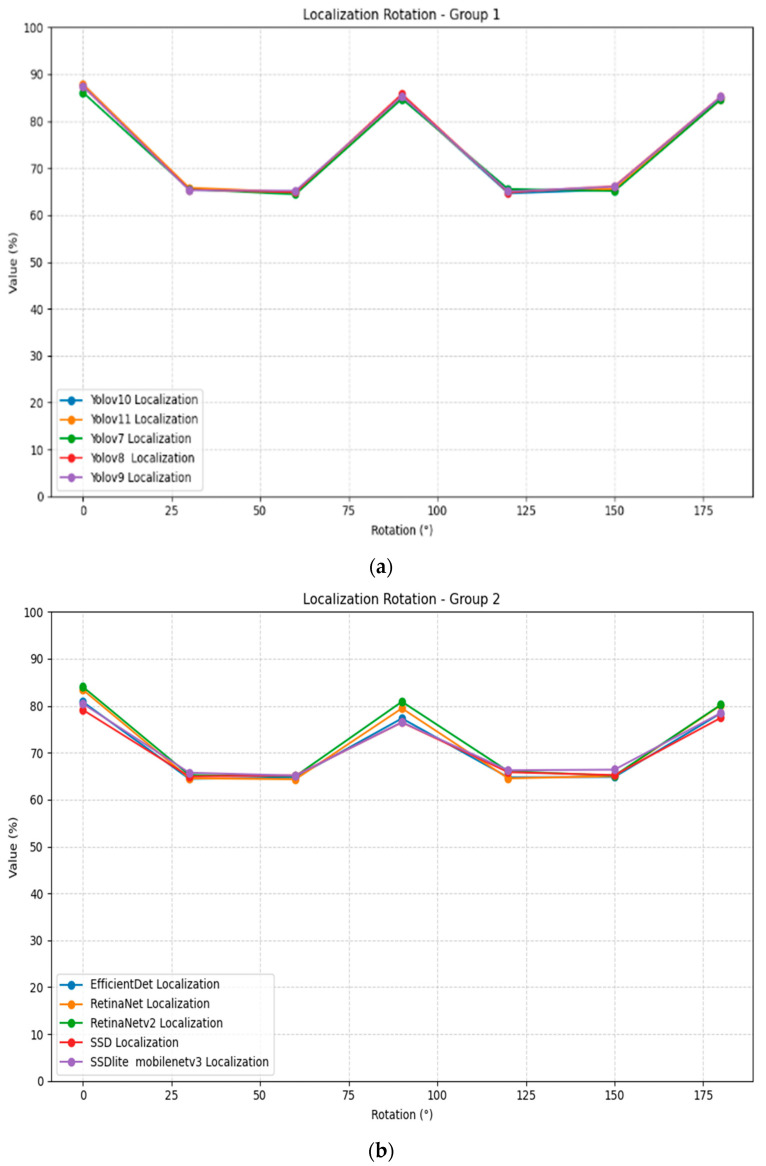
Localization rotation plots for all models and strengths: (**a**) Group 1; (**b**) Group 2.

Rotation transformation reveals significant invariance limitations across all localization models, as shown in Table 6. The results demonstrate a consistent pattern where performance at 90° and 180° rotation angles approaches baseline levels, while intermediate angles (30°, 60°, 120°, 150°) cause substantial performance degradation. This can be attributed to many possible factors, such as the visibility of the subject after the rotation. Figure 7 illustrates the localization rotation plots for all models, divided into two groups for better visualization.

**Figure 8:**
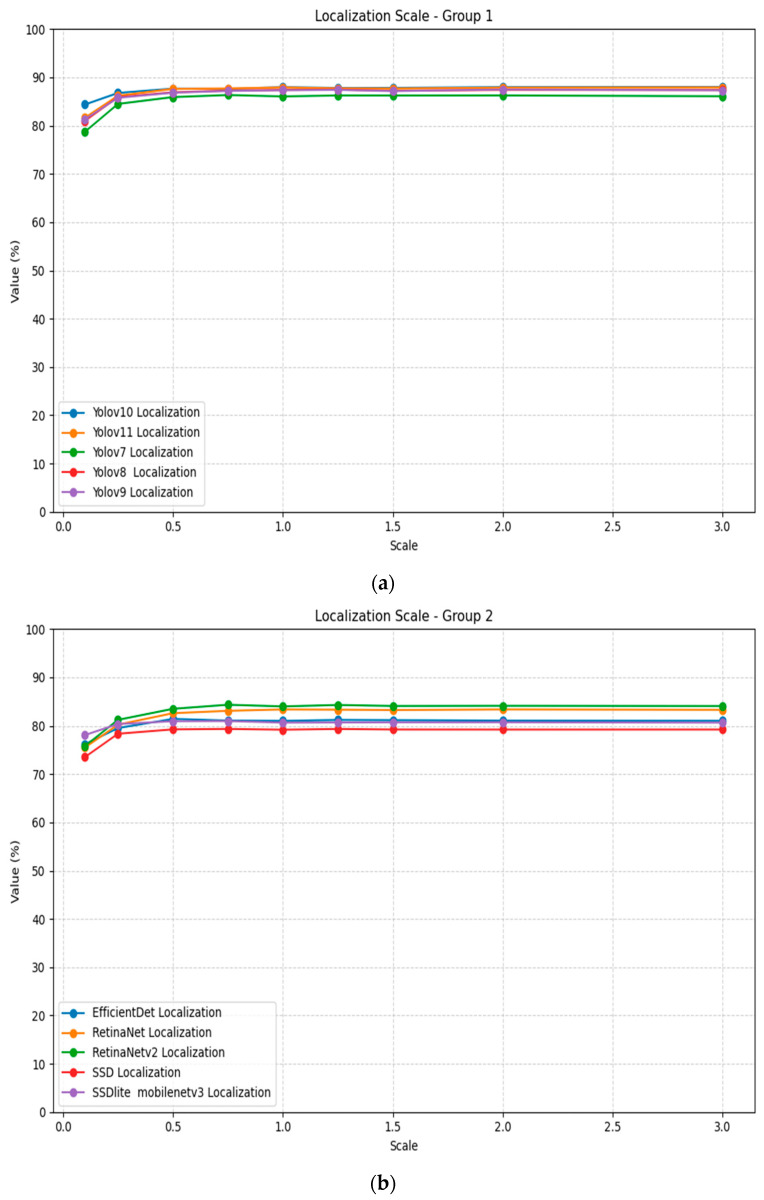
Localization scaling plots for all models and strengths: (**a**) Group 1; (**b**) Group 2.

Scale transformation demonstrateσ varying impact on localization performance, as presented in Table 7. Most models show degraded performance at extreme downscaling (0.1×) and some improvement as the scale approaches the baseline, while after that, staying near it. Figure 8 illustrates the localization scaling plots for all models, divided into two groups for better visualization.

### 5.2. Object Recognition

Recognition models demonstrate varying degrees of blur robustness across different architectures, as shown in Table 8. ConvNeXt achieves the highest baseline accuracy at 0.84 with σ = 0, followed by Swin Transformer at 0.82. Under increasing blur intensity, ConvNeXt maintains relatively strong performance compared to other models, declining to 0.60 at σ = 4, representing a 29% accuracy reduction. Vision Transformer shows superior blur robustness, declining from 0.81 to 0.62, demonstrating only a 24% performance loss.

**Figure 9:**
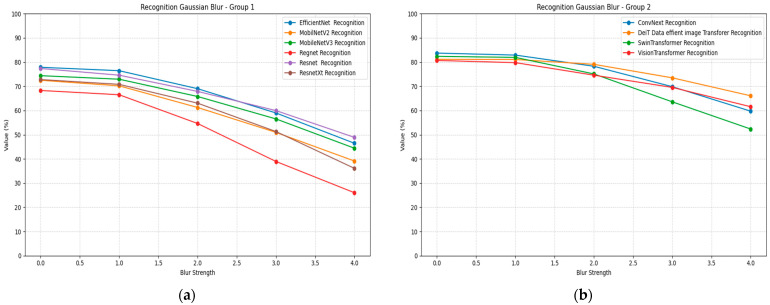
Recognition Gaussian blur plots for all models and strengths: (**a**) Group 1; (**b**) Group 2.

Mobile architectures exhibit significant blur sensitivity, with MobileNetV2 dropping from 0.73 to 0.39 accuracy and RegNet showing the most severe degradation from 0.68 to 0.26. EfficientNet demonstrates moderate blur robustness, maintaining 0.46 accuracy at σ = 4 from a baseline of 0.78. Swin Transformer, despite strong baseline performance, shows substantial blur sensitivity with accuracy declining to 0.52 at maximum blur intensity, showing that transformers also have trouble with the recognition of blurred images. Figure 9 illustrates the recognition blur plots for all models, divided into two groups for better visualization Group 1 includes EfficientNet, MobilNetV2, MobileNetV3, Regnet, Resnet and ResnetXt, while Group 2 includes ConvNext, DeiT-Base, SwinTransformer, and VisionTransformer.

Gaussian noise significantly impacts recognition accuracy across all architectures, as demonstrated in Table 9. The column headers appear to have some inconsistencies in the provided data, but the general pattern shows severe performance degradation with increasing noise levels. ConvNeXt shows remarkable noise robustness as opposed to the rest of the CNN models, declining from an accuracy of 0.84 to 0.32. All transformer models maintain strong noise robustness with the smallest performance decline among tested models.

**Figure 10:**
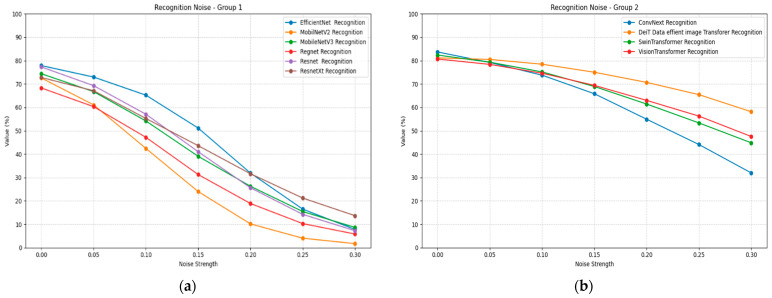
Recognition Gaussian noise plots for all models and strengths: (**a**) Group 1; (**b**) Group 2.

Mobile architectures demonstrate severe noise sensitivity, with MobileNetV2 showing near-complete failure under high noise conditions. EfficientNet exhibits substantial noise sensitivity despite its baseline performance. Vision Transformer shows moderate noise robustness, maintaining better performance than CNN-based architectures under equivalent noise conditions. Figure 10 illustrates the recognition noise plots for all models, divided into two groups for better visualization.

Rotation transformation reveals significant limitations in recognition model invariance, as shown in Table 10. All models exhibit substantial performance degradation at intermediate rotation angles while showing some recovery at 90° and 180° rotation angles. ConvNeXt maintains the most stable rotation performance, declining from 0.84 to approximately 0.70 to 0.77 at intermediate angles. Swin Transformer demonstrates superior rotation robustness, maintaining above 0.72 accuracy across most rotation angles.

**Figure 11:**
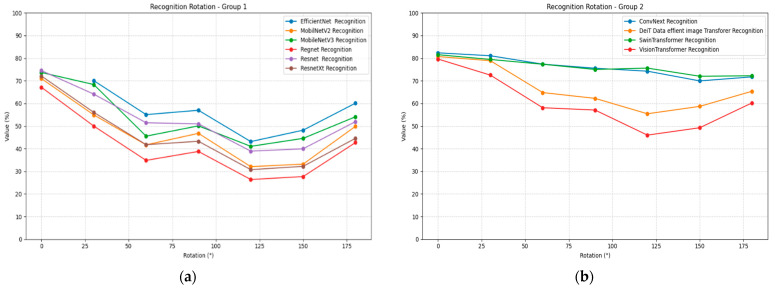
Recognition rotation plots for all models and strengths: (**a**) Group 1; (**b**) Group 2.

EfficientNet shows severe rotation sensitivity, with performance dropping to below 0.50 at many intermediate angles. Mobile architectures, once again, demonstrate significant vulnerability, with MobileNetV2 and MobileNetV3 showing substantial performance losses. Vision Transformer exhibits moderate rotation sensitivity but maintains better performance than most CNN architectures at equivalent rotation angles. Figure 11 illustrates the recognition performance under rotation plots for all models, divided into two groups for better visualization.

Scale transformation reveals significant architectural differences in invariance handling, as presented in Table 11. At extreme downscaling (0.1×), all models show substantial performance degradation, with RegNet performing worst at 0.13 accuracy and Deit showing the best resilience at 0.47. Performance generally improves as scale increases toward baseline resolution, with most models achieving optimal performance around 1.0× to 1.25× scaling.

**Figure 12:**
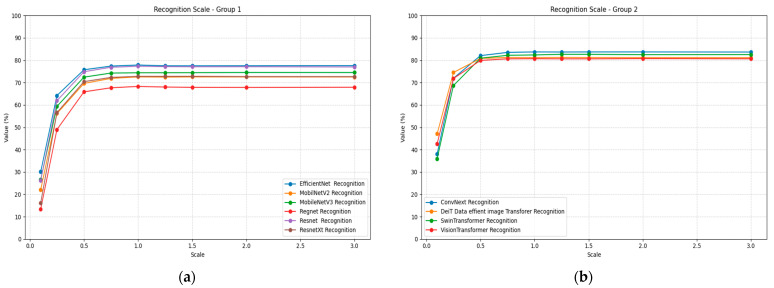
Recognition scaling plots for all models and strengths: (**a**) Group 1; (**b**) Group 2.

SwinTransformer demonstrates the most stable scaling performance, maintaining above 0.80 across the 0.75× to 2.0× range and achieving peak performance of 0.83 at 1.25× scaling. Vision Transformer shows similar stability with minimal performance variation across scale factors. Mobile architectures again show significant scale sensitivity, with MobileNetV2 and MobileNetV3 exhibiting substantial performance losses at small scale factors. Figure 12 illustrates the recognition performance under scaling plots for all models, divided into two groups for better visualization.

### 5.3. Semantic Segmentation

Segmentation models demonstrate varied blur robustness patterns, as presented in Table 12. Mask2Former achieves the highest baseline performance with 0.73 mIoU and maintains superior blur robustness, declining to 0.68 mIoU at σ = 4. CLIPSeg shows excellent blur stability, maintaining above 0.65 mIoU across all blur levels. DeepLabV3 variants demonstrate moderate blur sensitivity, with the ResNet-101 version maintaining 0.43 mIoU at maximum blur intensity.

**Figure 13:**
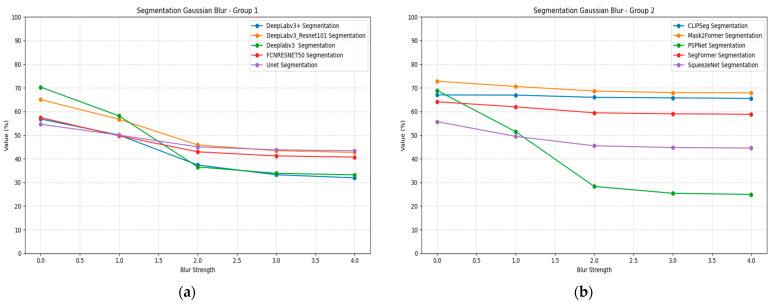
Segmentation Gaussian blur plots for all models and strengths: (**a**) Group 1; (**b**) Group 2.

PSPNet exhibits severe blur sensitivity, dropping from 0.69 to 0.25 mIoU, representing a 64% performance loss. SegFormer maintains good blur robustness with minimal performance degradation across blur levels. UNet and SqueezeNet show moderate blur sensitivity, with performance declining to approximately 0.43–0.45 mIoU at σ = 4. Figure 13 illustrates the segmentation performance under blurring plots for all models, divided into two groups for better visualization. Group 1 includes DeepLabv3+, DeepLabv3_Resnet101, Deeplabv3, FCN, and Unet, while Group 2 includes CLIPSeg, Mask2Former, PSPNet, SegFormer, and SqueezeNet.

Gaussian noise significantly impacts segmentation performance across all tested models, as shown in Table 13. Mask2Former demonstrates the most robust noise handling, declining from 0.73 to 0.35 mIoU but maintaining the highest absolute performance under low noise conditions, while being surpassed by the more stable SegFormer model. CLIPSeg shows good noise robustness, maintaining 0.39 mIoU at σ = 0.3. SegFormer exhibits stable noise performance with gradual degradation across noise levels.

**Figure 14:**
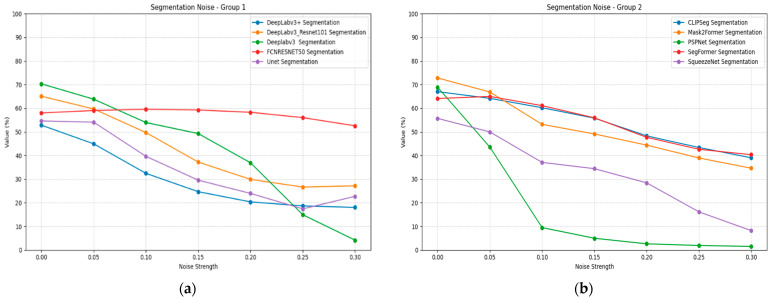
Segmentation Gaussian noise plots for all models and strengths: (**a**) Group 1; (**b**) Group 2.

PSPNet shows severe noise sensitivity, with performance dropping to 0.02 mIoU at maximum noise intensity. FCN ResNet-50 demonstrates an unusual pattern, initially showing slight performance improvement at low noise levels before degrading at higher intensities. DeepLabV3 variants show substantial noise sensitivity, with the standard version performing worse than the ResNet-101 variant. Figure 14 illustrates the segmentation performance under noise plots for all models, divided into two groups for better visualization.

Rotation transformation affects segmentation models differently than localization and recognition tasks, as demonstrated in Table 14. Most segmentation models show relatively stable performance across rotation angles, with performance variations typically within 0.05–0.10 mIoU. The DeepLabv3 models maintain strong rotation robustness, showing minimal performance variation across all tested angles. CLIPSeg demonstrates excellent rotation stability with consistent performance around 0.59–0.67 mIoU.

**Figure 15:**
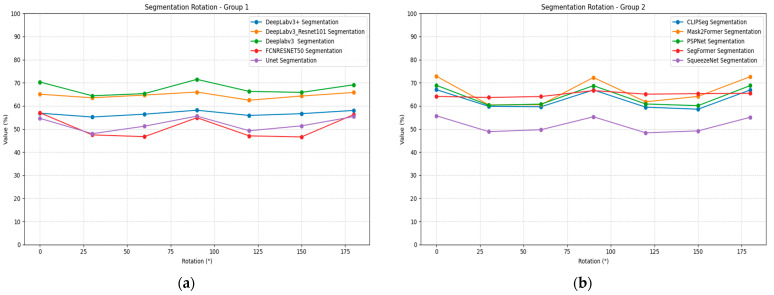
Segmentation rotation plots for all models and strengths: (**a**) Group 1; (**b**) Group 2.

PSPNet maintains consistent performance across rotation angles, suggesting good rotational invariance properties. SegFormer shows minimal rotation sensitivity with performance remaining stable across all tested angles. Figure 15 illustrates the segmentation performance under rotation plots for all models, divided into two groups for better visualization.

Scale transformation reveals significant architectural differences in segmentation model robustness, as presented in Table 15. At extreme downscaling (0.1×), most models show substantial performance degradation, with several models achieving below 0.10 mIoU. Mask2Former demonstrates the most robust scaling performance, maintaining above 0.31 mIoU even at 0.1× scale and achieving peak performance of 0.729 mIoU at 1.25× scaling.

**Figure 16:**
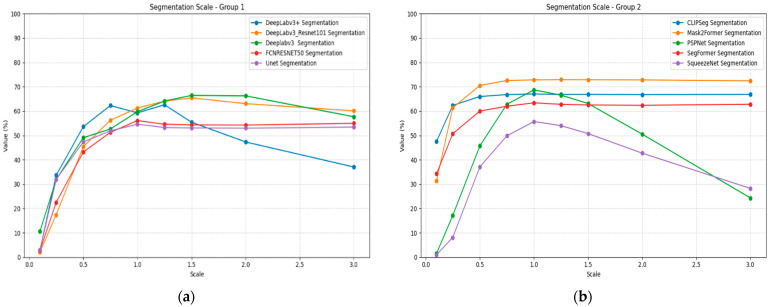
Segmentation scale plots for all models and strengths: (**a**) Group 1; (**b**) Group 2.

CLIPSeg shows good scale robustness, maintaining above 0.47 mIoU at 0.1× scale and stable performance across larger scale factors. SegFormer exhibits moderate scale sensitivity with substantial performance loss at small scales but good recovery at normal and large scales. PSPNet, DeepLabv3 with Resenet101, DeepLabc3+ and SqueezeNet show severe scale sensitivity, with near-zero performance at 0.1× scaling. Figure 16 illustrates the segmentation performance under scaling plots for all models, divided into two groups for better visualization.

The empirical results reveal several consistent patterns across model architectures and tasks. Vision Transformers (ViT, Swin Transformer, DeiT) generally demonstrate superior robustness to blur and noise transformations compared to CNN architectures, particularly in recognition tasks. However, rotation invariance remains challenging for all architecture types, with performance degradation occurring at intermediate angles regardless of model family.

Scale invariance exhibits the most dramatic performance variations, with extreme downscaling (0.1×) resulting in near-complete failure in many models across all tasks. Modern YOLO variants demonstrate relatively stable performance across most invariance types in localization tasks, while mobile architectures consistently show the highest sensitivity to all transformation types. SegFormer emerges as the most robust segmentation model across all invariance types, as its delta on performance decrease is the lowest among the models. In contrast, traditional CNN-based segmentation approaches show significant sensitivity to most transformations.

### 5.4. Case Studies of Misclassifications Across Architectures

To complement the quantitative results, we include qualitative examples that illustrate typical failure cases for each of the three deep model usages evaluated in this study. These examples provide insight into how and why certain models struggle with specific transformations or object features. Each case shows the ground truth label alongside the model’s incorrect prediction, before applying any transformation. These indicative cases were selected to highlight common patterns in the types of errors made by each architecture.

**Figure 17:**
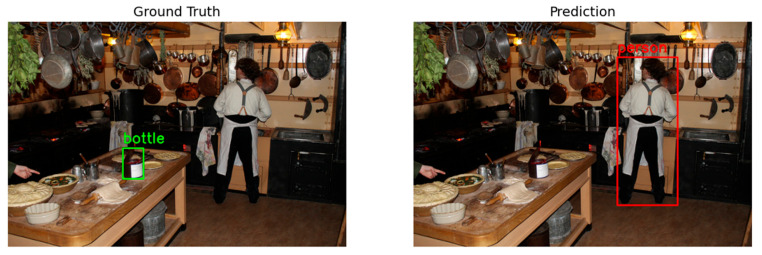
Indicative examples of object localization incorrect predictions with Yolov8 using an unprocessed image from the COCO dataset. Columns from left to right: ground truth, and incorrect prediction.

Regarding the localization models an interesting observation was made. The COCO dataset generally consists of images that depict multiple objects in the scene, as shown in Figure 17. The issue here lies with the labeling, as for each picture, certain aspects of the image are labeled as for example, in the case of the left image of Figure 17, the bottle. However, in this case, the model selects the person, which is not completely wrong, yet the prediction is mismatched.

**Figure 18:**
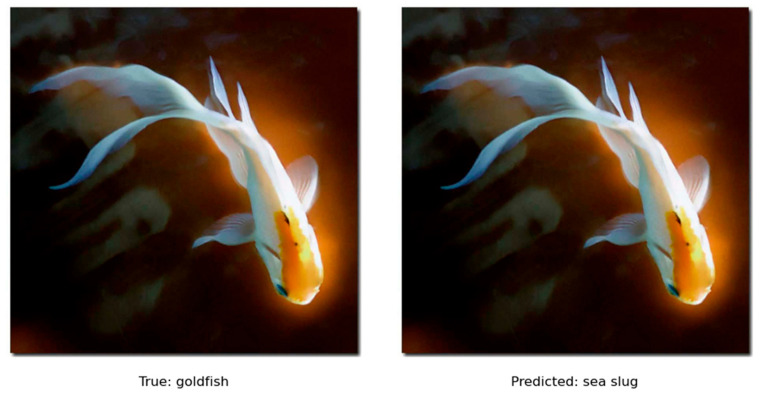
Indicative examples of object recognition incorrect predictions with SwinTransformer using an unprocessed image from the ImageNet dataset. Columns from left to right: ground truth, and incorrect prediction.

The recognition models provide more simple results. It was observed that many predictions by the models misclassified labels had similar properties. For example, in Figure 18, the ground truth image shows a goldfish, and the model predicted a sea slug. Therefore, the model can detect similar animals (i.e., fish) but sometimes lacks the fine-grained detection of the subject.

**Figure 19:**
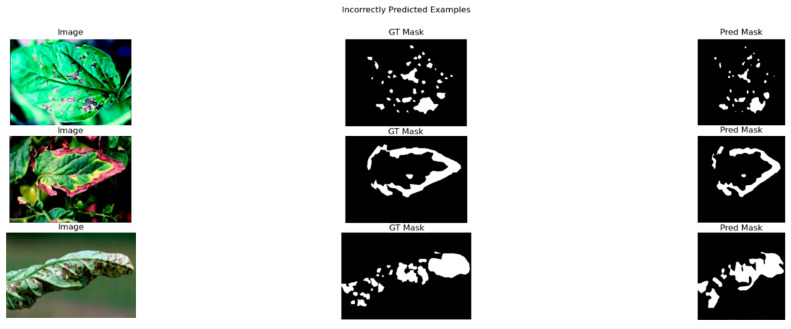
Indicative examples of semantic segmentation incorrect predictions with Mask2Former using an unprocessed image from the Leaf disease segmentation dataset. Columns from left to right: input image, ground truth mask, and incorrect prediction masks.

For the segmentation prediction in Figure 19, it can be seen that the model can’t completely predict the original masks of the leaves, yet it manages to simulate their major part.

These qualitative examples reinforce the quantitative results, showing that even the best-performing models have specific weaknesses. Such visualizations aim to clarify the limitations of current architectures and motivate further research towards improving model robustness across diverse input variations. It should be noted that many observed differences between models—often within ±0.01 mAP or mIoU—are smaller than the expected statistical noise from the limited test set and should be interpreted as indicative trends rather than definitive claims of model superiority.

## 6. Discussion

### 6.1. Observation Points

The empirical results reveal fundamental architectural trade-offs in handling different invariance types across vision tasks. While YOLO variants demonstrated exceptional blur and scale robustness in localization under noise, their results expose vulnerabilities in the frequency-domain. This aligns with the theoretical observations from Azulay et al. [74], who attributed CNNs’ sensitivity to improper sampling of high-frequency components. The counterintuitive SSD performance improvement under moderate blur suggests blur-induced regularization effects, potentially mitigating overfitting to high-frequency artifacts as hypothesized in Cui et al.’s anti-aliasing work [75].

Vision Transformers exhibited superior noise robustness, supporting the findings of Wang et al. [76] of ViTs’ reduced high-frequency sensitivity. However, their rotation performance parity with CNNs challenges assumptions about attention mechanisms inherently solving geometric invariance, corroborating recent debates in the work of Pinto et al. [77]. The segmentation results further enhance this narrative that shows that transformers overall seem to have broadly a more stable performance, even in higher strengths of different image degradations.

Our multi-task analysis uncovered a fundamental scale invariance paradox: while localization models maintained their functionality at 0.1× scaling, recognition and segmentation models suffered catastrophic failures. This dichotomy suggests scale invariance operates through distinct mechanisms in detection versus classification architectures. The localization results support the feature pyramid hypothesis of Mumuni et al. [78], where multi-scale anchors provide inherent scale resilience.

The universal rotation sensitivity across architectures underscores a critical limitation of modern deep vision systems. The segmentation exception suggests dense prediction tasks may indirectly learn rotation-equivariant features through pixel-wise consistency objectives, which is a phenomenon meriting further investigation.

The noise results expose a fundamental CNN-ViT divergence: while CNNs suffered progressive degradation, ViTs displayed thresholded failure modes. The segmentation anomaly (FCN ResNet-50’s increase in mIoU at σ = 0.05 as seen in Table 13) parallels biomedical imaging findings where low noise regularizes over-segmentation [79], suggesting task-dependent noise responses.

### 6.2. Limitations and Future Directions

While comprehensive, this study faces specific limitations, summarized in the following points:

1. Static Transformation Analysis: Real-world invariances often involve combined perturbations, which are absent from our isolated tests.
2. Simplicity of Transformations: The applied perturbations (blur, noise, rotation, scale) were simulated independently and represent clean, idealized distortions. In practice, corruptions frequently co-occur (e.g., blur + rotation, scale + occlusion) or appear partially across the image, producing more complex challenges. Evaluating such compounded perturbations remains an important direction for future work. Yet, it should be noted that isolating individual transformations allows for a more precise evaluation of the models’ sensitivity and robustness to specific types of perturbations, while single transformations in this work also aim to serve as a clear baseline of a foundational benchmark.
3. Dataset Bias: COCO/ImageNet focus limits ecological validity for specialized domains like medical or satellite imaging.
4. Black-Box Metrics: Layer-wise invariance analysis could reveal mechanistic insights beyond task performance.
5. Given our modest per-class sample size, marginal score differences (e.g., ±0.01 in mAP or mIoU) are not necessarily statistically significant and should not be over-interpreted.
6. Limited computational resources: in this work subsets of data were considered to fine-tune the segmentation pre-trained models, mainly due to limited available resources. Considering recent robustness benchmarks specifically designed for object detection, such as ImageNet-C Hendrycks and Dietterich [41], future work will aim enhance the size of used datasets.

Recent advances in complex and dynamic environments pose significant challenges for testing the invariance capabilities of vision models. In surgical contexts, Zhang et al. [80] proposed adaptive graph learning frameworks that anticipate surgical workflow by modeling dynamic interactions under varying factors such as lighting changes, occlusions, and motion-induced artifacts typical in robotic-assisted procedures. These settings introduce compounded invariance challenges involving simultaneous blur, scale variations, and geometric transformations, thereby providing realistic benchmarks for assessing model robustness in localization and segmentation tasks critical to patient safety. Similarly, in underwater detection scenarios, Ge et al. [81] presented datasets capturing marine organisms under diverse environmental conditions like illumination changes, turbidity, and color distortion. These factors inherently combine noise, blur, color shifts, and scale variations, offering an ecologically valid framework to evaluate model robustness under naturally occurring complex perturbations. Both domains underscore the necessity of evaluating vision models beyond isolated invariance tests to reflect real-world, multi-faceted transformation challenges.

The systematic evaluation of models across such challenging domains would provide more actionable insights for real-world deployment. Surgical tool detection in dynamic scenarios and underwater object recognition represent domains where invariance failures can have significant consequences—patient safety in surgical robotics and autonomous underwater vehicle navigation, respectively. Therefore, future work should prioritize evaluation on these challenging datasets to better understand the practical limitations of current deep learning architectures.

Deep learning models exhibit significant vulnerabilities to fundamental image transformations. Systematic evaluation of thirty CNNs and ViTs across localization, recognition, and segmentation tasks reveals that while ViTs outperform CNNs under blur and noise in recognition, both architectures suffer substantial degradation under rotation and extreme scale transformations. Segmentation models, particularly SegFormer and Mask2Former, demonstrate superior geometric robustness. These findings expose persistent architectural limitations and underscore the need for explicit invariance mechanisms in vision systems [41].

Regarding the Scale Invariance Paradox, its observation suggests that multi-scale anchor mechanisms in detection architectures provide inherent scale resilience absent in classification systems. Potential solutions include: (1) integrating feature pyramid networks (FPNs) into recognition and segmentation architectures to enable multi-scale processing; (2) implementing scale-equivariant convolutions that explicitly preserve scale relationships across network layers; (3) adopting pyramid pooling strategies from localization models; and (4) developing hybrid training protocols that expose classification models to extreme scale variations during pre-training, mimicking the multi-scale robustness observed in detection frameworks.

These results collectively challenge the notion of universal architectural superiority, instead advocating for task-specific model selection guided by operational invariance requirements. The findings particularly underscore the urgent need for standardized robustness benchmarks beyond conventional accuracy metrics in real-world vision system deployment.

## 7. Conclusions

This empirical study provides comprehensive evidence of significant variation in robustness patterns across deep learning architectures and transformation types, challenging assumptions about inherent invariance capabilities in modern vision systems. The systematic evaluation of 30 models across three computer vision tasks reveals fundamental limitations that persist across architectural families, with implications for real-world deployment of deep vision systems.

### 7.1. Key Empirical Findings

Results demonstrate that Vision Transformers consistently outperformed CNN architectures in handling blur and noise transformations across recognition tasks. Vision Transformer exhibited lower performance loss under maximum blur intensity (σ = 4), compared to substantially higher degradation in CNN-based models such as RegNet, which showed extreme performance loss under equivalent conditions. Similarly, noise robustness analysis revealed that ViTs maintained better performance retention compared to CNN architectures under equivalent noise conditions. In general, all models show signs of performance degradation when applying noise and blur, regardless of their underlying architecture.

All tested architectures, regardless of their type (CNN or Transformer), exhibited substantial performance degradation at intermediate rotation angles (30°, 60°, 120°, 150°) while showing recovery at 90° and 180° rotations. This pattern emerged consistently across localization, recognition, and segmentation tasks, while for the segmentation models, the degradation was smaller, indicating that rotation invariance remains an unsolved challenge for current deep learning architectures.

Extreme downscaling (0.1×) caused near-complete failure across all model types and tasks, with recognition and segmentation systems suffering more severe degradation than localization models. This scale invariance paradox reveals fundamental differences in how various vision tasks handle scale transformations, with localization models maintaining some functionality where classification systems fail completely.

These findings represent two novel empirical contributions: (1) the scale invariance paradox demonstrates fundamental architectural differences in handling extreme transformations across vision tasks, challenging assumptions about unified robustness; and (2) ViT thresholded failure modes under noise reveal discrete rather than gradual degradation patterns, contrasting with CNN progressive failure and suggesting different underlying robustness mechanisms.

### 7.2. Task-Specific Invariance Patterns

The multi-task analysis revealed distinct invariance mechanisms operating across computer vision applications. Segmentation models demonstrated superior rotation stability compared to localization and recognition tasks, with most models showing performance variations within 0.05–0.10 mIoU across rotation angles. This suggests that dense prediction tasks may inherently develop rotation-equivariant features through pixel-wise consistency objectives.

Localization models maintained functionality under extreme scale transformations where recognition systems failed, supporting the hypothesis that multi-scale anchor mechanisms provide inherent scale resilience in detection architectures. The noise robustness analysis exposed fundamental CNN-ViT divergences, with CNNs suffering progressive degradation while ViTs displayed thresholded failure modes.

The documented vulnerabilities across all tested architectures underscore the urgent need for developing dedicated invariance mechanisms beyond conventional data augmentation strategies. The superior performance of certain models under specific transformation types (e.g., Mask2Former for segmentation, Vision Transformers for noise robustness) provides empirical guidance for practitioners selecting models based on expected operational conditions.

### 7.3. Future Research Directions

This study establishes a foundation for several critical research directions. The development of standardized robustness benchmarks beyond conventional accuracy metrics emerges as an immediate priority for the computer vision community. Future investigations should examine combined transformation effects and explore layer-wise invariance analysis to reveal mechanistic insights into architectural differences.

The documented scale invariance paradox and rotation sensitivity across all architectures indicate fundamental theoretical gaps that require novel architectural innovations rather than incremental improvements. The task-specific invariance patterns observed suggest potential for developing hybrid architectures that leverage the strengths of different model families for enhanced robustness across transformation types.

The empirical evidence presented in this study contributes to the growing body of literature documenting the limitations of current deep learning approaches while providing quantitative benchmarks for evaluating future invariance-aware architectures. Our findings emphasize that achieving robust vision systems requires explicit consideration of invariance properties during both architectural design and model selection phases, moving beyond the assumption that deeper or larger models inherently provide superior robustness capabilities.

## Figures and Tables

**Table 1:** Details of selected models for object localization.

Model	Short Name	Description	Date Introduced	Architecture	Dataset
EfficientDet-D0	EfficientDet	Lightweight detector using EfficientNet backbone and BiFPN	2019	CNN	COCO 2017
ResNet-50 backbone + FPN + RetinaNet head (Focal Loss)	RetinaNet	Single-stage detector with a ResNet-50-FPN backbone and Focal Loss to address class imbalance	2017	CNN	COCO 2017
ResNet-50 backbone + FPN + RetinaNet head (ATSS) with ResnetV2	RetinaNetv2	Improved RetinaNet with a ResNet-50-FPN backbone incorporating Adaptive Training Sample Selection (ATSS)	2019	CNN	COCO 2017
SSD300 VGG16	SSD	Single Shot MultiBox Detector with VGG16 backbone, fast and lightweight	2016	CNN	COCO 2017
MobileNetV3-Large backbone + SSD-Lite head	SSDlite	Single-Shot Detector Lite with a MobileNetV3-Large backbone for fast, efficient object detection	2019	CNN	COCO 2017
YOLOv10	Yolov10	Latest iteration with focus on real-time performance and transformer enhancements	2024	CNN	COCO 2017
YOLOv11	Yolov11	Introduces advanced feature fusion and sparse attention mechanisms	2024	CNN	COCO 2017
YOLOv7	Yolov7	Improved accuracy and speed over v5, optimized for diverse tasks, including pose estimation	2022	CNN	COCO 2017
YOLOv8	Yolov8	Modular and extensible, supports classification, segmentation, and detection	2023	CNN	COCO 2017
YOLOv9	Yolov9	Introduces faster training and inference with hardware-aware optimization	2024	CNN	COCO 2017

**Table 2:** Details of selected models for object recognition.

Model	Short Name	Description	Date Introduced	Architecture	Dataset
EfficientNet-B0	EfficientNet	Compound-scaled CNN with great efficiency and accuracy trade-off	2019	CNN	ILSVRC2012
MobileNetV2	MobilNetV2	Lightweight CNN optimized for mobile inference	2018	CNN	ILSVRC2012
MobileNetV3-Large	MobileNetV3	Optimized version of MobileNetV2 using NAS and SE blocks	2019	CNN	ILSVRC2012
RegNetY-400MF	Regnet	Regularized network design with performance/efficiency trade-off	2020	CNN	ILSVRC2012
ResNet-50	Resnet	50-layer deep CNN with skip connections, strong baseline	2015	CNN	ILSVRC2012
ResNeXt-50 (32x4d)	ResnetXt	Improved ResNet variant with grouped convolutions	2017	CNN	ILSVRC2012
ConvNeXt-Base	ConvNext	CNN redesigned with transformer-like properties for SOTA performance	2022	CNN	ILSVRC2012
DeiT-Base Patch16/224	DeiT-Base	Data-efficient vision transformer trained without large datasets	2021	Transformer	ILSVRC2012
Swin Transformer Base	SwinTransformer	Shifted window transformer with hierarchical vision transformer design	2021	Transformer	ILSVRC2012
Vision Transformer (ViT-B/16)	VisionTransformer	Vision Transformer with 16x16 patch embedding, no CNNs	2020	Transformer	ILSVRC2012

**Table 3:** Details of selected models for segmentation.

Model	Short Name	Description	Date Introduced	Architecture	Dataset
DeepLabV3+ (ResNet-101)	DeepLabv3+	Dilated convolution with encoder-decoder refinement using ResNet-101	2018	CNN	Custom Leaf Dataset
DeepLabV3 (ResNet-101)	DeepLabv3_Resnet101	Atrous spatial pyramid pooling with ResNet-101 backbone	2017	CNN	Custom Leaf Dataset
DeepLabV3 (ResNet-50)	Deeplabv3	Smaller version of DeepLabV3 using ResNet-50 as encoder	2017	CNN	Custom Leaf Dataset
FCN (ResNet-50)	FCN	Fully convolutional network using ResNet-50 as backbone	2015	CNN	Custom Leaf Dataset
UNet (ResNet-34)	Unet	UNet encoder-decoder architecture with ResNet-34 as backbone	2015	CNN	Custom Leaf Dataset
CLIPSeg	CLIPSeg	CLIP-based model for text-prompted image segmentation	2022	Transformer	Custom Leaf Dataset
Mask2Former	Mask2Former	Universal transformer-based model for semantic, instance, and panoptic segmentation	2022	Transformer	Custom Leaf Dataset
PSPNet (ResNet-50)	PSPNet	Pyramid Scene Parsing Network with ResNet-50 encoder backbone	2017	CNN	Custom Leaf Dataset
SegFormer	SegFormer	Transformer-based semantic segmentation model, efficient and accurate	2021	Transformer	Custom Leaf Dataset
SqueezeNet 1.1	SqueezeNet	Tiny CNN for classification, sometimes used in lightweight segmentation	2016	CNN	Custom Leaf Dataset

**Table 4:** Models’ robustness evaluation (mAP) on object localization under Gaussian blur transformation of different strengths.

Model	Gaussian Blur of Different Strengths (Sigma)				
	0	1	2	3	4
EfficientDet	0.81	0.81	0.81	0.81	0.81
RetinaNet	0.83	0.83	0.83	0.82	0.82
RetinaNetv2	0.84	0.84	0.83	0.83	0.83
SSD	0.80	0.79	0.80	0.80	0.80
SSDlite	0.81	0.81	0.80	0.81	0.81
Yolov10	0.88	0.88	0.87	0.87	0.87
Yolov11	0.88	0.88	0.87	0.87	0.87
Yolov7	0.86	0.86	0.86	0.86	0.86
Yolov8	0.87	0.87	0.87	0.87	0.87
Yolov9	0.87	0.87	0.87	0.87	0.87

**Table 5:** Models’ robustness evaluation (mAP) on object localization under Gaussian noise of different strengths.

Model	Gaussian Noise of Different Strengths (std)						
	0	0.05	0.1	0.15	0.2	0.25	0.3
EfficientDet	0.81	0.81	0.80	0.79	0.79	0.78	0.78
RetinaNet	0.83	0.82	0.82	0.81	0.80	0.79	0.78
RetinaNetv2	0.84	0.84	0.83	0.81	0.80	0.77	0.75
SSD	0.80	0.80	0.79	0.79	0.79	0.78	0.77
SSDlite	0.81	0.81	0.81	0.80	0.80	0.80	0.81
Yolov10	0.88	0.87	0.86	0.85	0.85	0.83	0.83
Yolov11	0.88	0.87	0.86	0.85	0.84	0.83	0.82
Yolov7	0.86	0.85	0.85	0.83	0.83	0.83	0.81
Yolov8	0.87	0.86	0.85	0.85	0.84	0.84	0.83
Yolov9	0.87	0.86	0.86	0.85	0.84	0.83	0.82

**Table 6:** Models’ robustness evaluation (mAP) on object localization under rotation transformation of different angles.

Model	Rotation of Different Angles (Angle)						
	0°	30°	60°	90°	120°	150°	180°
EfficientDet	0.81	0.64	0.65	0.77	0.65	0.65	0.78
RetinaNet	0.83	0.65	0.64	0.79	0.65	0.65	0.80
RetinaNetv2	0.84	0.65	0.65	0.81	0.66	0.65	0.80
SSD	0.80	0.65	0.65	0.76	0.66	0.65	0.77
SSDlite	0.81	0.66	0.65	0.77	0.66	0.66	0.78
Yolov10	0.88	0.65	0.65	0.85	0.65	0.65	0.85
Yolov11	0.88	0.66	0.65	0.85	0.65	0.66	0.85
Yolov7	0.86	0.65	0.64	0.85	0.66	0.65	0.85
Yolov8	0.87	0.65	0.65	0.86	0.65	0.66	0.85
Yolov9	0.87	0.65	0.65	0.85	0.65	0.66	0.85

**Table 7:** Models’ robustness evaluation (mAP) on object localization under scaling transformation of different scales.

Model	Scaling of Different Factors (Scale)								
	0.1	0.25	0.5	0.75	1	1.25	1.5	2	3
EfficientDet	0.76	0.80	0.81	0.81	0.81	0.81	0.81	0.81	0.81
RetinaNet	0.76	0.80	0.83	0.83	0.83	0.83	0.83	0.83	0.83
RetinaNetv2	0.76	0.81	0.84	0.84	0.84	0.84	0.84	0.84	0.84
SSD	0.74	0.78	0.79	0.79	0.80	0.79	0.79	0.79	0.79
SSDlite	0.78	0.80	0.81	0.81	0.81	0.81	0.81	0.81	0.81
Yolov10	0.84	0.87	0.88	0.88	0.88	0.88	0.88	0.88	0.88
Yolov11	0.82	0.86	0.88	0.88	0.88	0.88	0.88	0.88	0.88
Yolov7	0.79	0.84	0.86	0.86	0.86	0.86	0.86	0.86	0.86
Yolov8	0.81	0.86	0.87	0.87	0.87	0.87	0.87	0.87	0.87
Yolov9	0.81	0.86	0.87	0.87	0.87	0.87	0.87	0.87	0.87

**Table 8:** Models’ robustness evaluation (Accuracy) on object recognition under Gaussian blur transformation of different strengths.

Model	Gaussian Blur of Different Strengths (Sigma)				
	0	1	2	3	4
EfficientNet	0.78	0.76	0.69	0.59	0.46
MobilNetV2	0.73	0.70	0.61	0.51	0.39
MobileNetV3	0.74	0.73	0.66	0.57	0.44
Regnet	0.68	0.67	0.55	0.39	0.26
Resnet	0.77	0.75	0.68	0.60	0.49
ResnetXt	0.73	0.71	0.63	0.51	0.36
ConvNext	0.84	0.83	0.78	0.70	0.60
DeiT-Base	0.81	0.81	0.79	0.74	0.66
SwinTransformer	0.82	0.82	0.75	0.64	0.52
VisionTransformer	0.81	0.80	0.75	0.70	0.62

**Table 9:** Models’ robustness evaluation (Accuracy) on object recognition under Gaussian noise of different strengths.

Model	Gaussian Noise of Different Strengths (std)						
	0	0.05	0.1	0.15	0.2	0.25	0.3
EfficientNet	0.78	0.73	0.65	0.51	0.32	0.16	0.08
MobilNetV2	0.73	0.61	0.42	0.24	0.10	0.04	0.02
MobileNetV3	0.74	0.67	0.54	0.39	0.26	0.15	0.09
Regnet	0.68	0.60	0.47	0.31	0.19	0.10	0.06
Resnet	0.77	0.69	0.57	0.41	0.26	0.14	0.07
ResnetXt	0.73	0.67	0.55	0.44	0.32	0.21	0.14
ConvNext	0.84	0.79	0.74	0.66	0.55	0.44	0.32
DeiT-Base	0.81	0.81	0.78	0.75	0.71	0.65	0.58
SwinTransformer	0.82	0.79	0.75	0.69	0.61	0.53	0.45
VisionTransformer	0.81	0.78	0.75	0.69	0.63	0.56	0.48

**Table 10:** Models’ robustness evaluation (Accuracy) on object recognition under rotation transformation of different angles.

Model	Rotation of Different Angles (Angle)						
	0°	30°	60°	90°	120°	150°	180°
EfficientNet	0.78	0.70	0.55	0.57	0.43	0.48	0.60
MobilNetV2	0.73	0.55	0.42	0.47	0.32	0.33	0.50
MobileNetV3	0.74	0.68	0.46	0.50	0.41	0.45	0.54
Regnet	0.68	0.50	0.35	0.39	0.26	0.28	0.43
Resnet	0.77	0.64	0.51	0.51	0.39	0.40	0.52
ResnetXt	0.73	0.56	0.42	0.43	0.31	0.32	0.45
ConvNext	0.84	0.81	0.77	0.76	0.74	0.70	0.72
DeiT-Base	0.81	0.79	0.65	0.62	0.55	0.59	0.65
SwinTransformer	0.82	0.79	0.77	0.75	0.76	0.72	0.72
VisionTransformer	0.81	0.73	0.58	0.57	0.46	0.49	0.60

**Table 11:** Models’ robustness evaluation (Accuracy) on object recognition under scaling transformation of different scales.

Model	Scaling of Different Factors (Scale)								
	0.1	0.25	0.5	0.75	1	1.25	1.5	2	3
EfficientNet	0.30	0.64	0.76	0.77	0.78	0.78	0.78	0.78	0.78
MobilNetV2	0.22	0.56	0.70	0.72	0.73	0.72	0.73	0.73	0.73
MobileNetV3	0.27	0.59	0.72	0.74	0.74	0.74	0.74	0.75	0.75
Regnet	0.13	0.49	0.66	0.68	0.68	0.68	0.68	0.68	0.68
Resnet	0.26	0.62	0.75	0.77	0.77	0.77	0.77	0.77	0.77
ResnetXt	0.16	0.57	0.70	0.72	0.73	0.73	0.73	0.73	0.73
ConvNext	0.38	0.72	0.82	0.84	0.84	0.84	0.84	0.84	0.84
DeiT-Base	0.47	0.75	0.81	0.81	0.81	0.81	0.81	0.81	0.81
SwinTransformer	0.36	0.69	0.81	0.82	0.82	0.83	0.83	0.82	0.83
VisionTransformer	0.43	0.72	0.80	0.81	0.81	0.81	0.81	0.81	0.81

**Table 12:** Models’ robustness evaluation (mIoU) on semantic segmentation under Gaussian blur transformation of different strengths.

Model	Gaussian Blur of Different Strengths (Sigma)				
	0	1	2	3	4
DeepLabv3+	0.57	0.50	0.37	0.33	0.32
DeepLabv3_Resnet101	0.65	0.57	0.46	0.43	0.43
Deeplabv3	0.70	0.58	0.37	0.34	0.33
FCN	0.57	0.50	0.43	0.41	0.41
Unet	0.55	0.50	0.45	0.44	0.43
CLIPSeg	0.67	0.67	0.66	0.66	0.66
Mask2Former	0.73	0.71	0.69	0.68	0.68
PSPNet	0.69	0.51	0.28	0.25	0.25
SegFormer	0.64	0.62	0.59	0.59	0.59
SqueezeNet	0.56	0.49	0.46	0.45	0.45

**Table 13:** Models’ robustness evaluation (mIoU) on semantic segmentation under Gaussian noise of different strengths.

Model	Gaussian Noise of Different Strengths (std)						
	0	0.05	0.1	0.15	0.2	0.25	0.3
DeepLabv3+	0.53	0.45	0.32	0.25	0.20	0.19	0.18
DeepLabv3_Resnet101	0.65	0.60	0.50	0.37	0.30	0.27	0.27
Deeplabv3	0.70	0.64	0.54	0.49	0.37	0.15	0.04
FCN	0.58	0.59	0.60	0.59	0.58	0.56	0.53
Unet	0.55	0.54	0.40	0.30	0.24	0.17	0.23
CLIPSeg	0.67	0.64	0.60	0.56	0.48	0.43	0.39
Mask2Former	0.73	0.67	0.53	0.49	0.44	0.39	0.35
PSPNet	0.69	0.44	0.09	0.05	0.03	0.02	0.02
SegFormer	0.64	0.65	0.61	0.56	0.48	0.43	0.40
SqueezeNet	0.56	0.50	0.37	0.34	0.28	0.16	0.08

**Table 14:** Models’ robustness evaluation (mIoU) on semantic segmentation under rotation transformation of different angles.

Model	Rotation of Different Angles (Angle)						
	0°	30°	60°	90°	120°	150°	180°
DeepLabv3+	0.57	0.55	0.56	0.58	0.56	0.57	0.58
DeepLabv3_Resnet101	0.65	0.64	0.65	0.66	0.62	0.64	0.66
Deeplabv3	0.70	0.64	0.65	0.71	0.66	0.66	0.69
FCN	0.57	0.47	0.47	0.55	0.47	0.47	0.56
Unet	0.55	0.48	0.51	0.56	0.49	0.51	0.55
CLIPSeg	0.67	0.60	0.60	0.67	0.59	0.59	0.67
Mask2Former	0.73	0.60	0.61	0.72	0.62	0.64	0.73
PSPNet	0.69	0.60	0.61	0.69	0.61	0.60	0.69
SegFormer	0.64	0.64	0.64	0.67	0.65	0.65	0.65
SqueezeNet	0.56	0.49	0.50	0.55	0.48	0.49	0.55

**Table 15:** Models’ robustness evaluation (mIoU) on semantic segmentation under scaling transformation of different scales.

Model	Scaling of Different Factors (Scale)								
	0.1	0.25	0.5	0.75	1	1.25	1.5	2	3
DeepLabv3+	0.03	0.34	0.54	0.62	0.59	0.63	0.55	0.47	0.37
DeepLabv3_Resnet101	0.02	0.17	0.45	0.56	0.61	0.64	0.65	0.63	0.60
Deeplabv3	0.11	0.32	0.49	0.53	0.60	0.64	0.66	0.66	0.58
FCN	0.03	0.23	0.43	0.51	0.56	0.55	0.54	0.54	0.55
Unet	0.03	0.32	0.47	0.52	0.55	0.53	0.53	0.53	0.53
CLIPSeg	0.48	0.62	0.66	0.67	0.67	0.67	0.67	0.67	0.67
Mask2Former	0.31	0.61	0.71	0.73	0.73	0.73	0.73	0.73	0.72
PSPNet	0.02	0.17	0.46	0.63	0.69	0.67	0.63	0.51	0.24
SegFormer	0.34	0.51	0.60	0.62	0.63	0.63	0.63	0.62	0.63
SqueezeNet	0.01	0.08	0.37	0.50	0.56	0.54	0.51	0.43	0.28

## Data Availability

The data presented in this study refer to three datasets available in public domains: (1) COCO dataset available in GitHub at https://github.com/cocodataset/cocodataset.github.io (assessed on 8 August 2025), (2) ImageNet ILSVRC2012 dataset available at https://www.image-net.org/challenges/LSVRC/2012/ (assessed on 08 August 2025), (3) Leaf disease segmentation dataset available in Kaggle at https://www.kaggle.com/datasets/fakhrealam9537/leaf-disease-segmentation-dataset (assessed on 8 August 2025).

## Abbreviations

The following abbreviations are used in this manuscript:

CNNs	Convolutional Neural Networks
ViTs	Vision Transformers
COCO	Common Objects in Context
mIoU	Mean Intersection over Union
Acc	Accuracy
SIFT	Scale-Invariant Feature Transform
SVMs	Support Vector Machines
ASIFT	Affine-Scale-Invariant Feature Transform
SURF	Speeded-Up Robust Features
ORB	Oriented FAST and Rotated BRIEF
FAST	Features from Accelerated Segment Test
BRIEF	Binary Robust Independent Elementary Features
HOG	Histogram of Oriented Gradients
SPM	Spatial Pyramid Matching
DIAL	Domain Invariant Adversarial Learning
DAT	Domain-wise Adversarial Training
DCT	Discrete Cosine Transform
PyramidAT	Pyramid Adversarial Training
SP-ViT	Spatial Prior-enhanced Vision Transformers
STNs	Spatial Transformer Networks
RViT	Rotation Invariant Vision Transformer
AMR	Artificial Mental Rotation
SPP	Spatial Pyramid Pooling
RiT	Rotation Invariance Transformer
ILSVRC2012	ImageNet Large Scale Visual Recognition Challenge 2012
FPNs	Feature pyramid networks
